# Neural basis underlying the trait of attachment anxiety and avoidance revealed by the amplitude of low-frequency fluctuations and resting-state functional connectivity

**DOI:** 10.1186/s12868-021-00617-4

**Published:** 2021-02-23

**Authors:** Min Deng, Xing Zhang, Xiaoyan Bi, Chunhai Gao

**Affiliations:** 1 Faculty of Psychology, Southwest University, BeiBei District, Chongqing, 400715 China; 2 College of Teacher and Education, Honghe University, Mengzi District, Yunnan, 651199 China; 3 College of Education Science and Management, Yunnan Normal University, Kunming, 650500 Yunnan China; 4 Research Center of Brain and Cognitive Neuroscience, Liaoning Normal University, Dalian, 116029 Liaoning Province China

**Keywords:** Attachment anxiety, Attachment avoidance, Resting-state fMRI, The amplitude of low-frequency fluctuations, Functional connectivity

## Abstract

**Background:**

Attachment theory demonstrates that early attachment experience shapes internal working models with mental representations of self and close relationships, which affects personality traits and interpersonal relationships in adulthood. Although research has focused on brain structural and functional underpinnings to disentangle attachment styles in healthy individuals, little is known about the spontaneous brain activity associated with self-reported attachment anxiety and avoidance during the resting state.

**Methods:**

One hundred and nineteen individuals participated in the study, completing the Experience in Close Relationship scale immediately after an 8-min fMRI scanning. We used the resting-state functional magnetic resonance imaging (rs-fMRI) signal of the amplitude of low-frequency fluctuation and resting-state functional connectivity to identify attachment-related regions and networks.

**Results:**

Consequently, attachment anxiety is closely associated with the amplitude of low-frequency fluctuations in the right posterior cingulate cortex, over-estimating emotional intensity and exaggerating outcomes. Moreover, the functional connectivity between the posterior cingulate cortex and fusiform gyrus increases detection ability for potential threat or separation information, facilitating behavior motivation. The attachment avoidance is positively correlated with the amplitude of low-frequency fluctuation in the bilateral lingual gyrus and right postcentral and negatively correlated with the bilateral orbital frontal cortex and inferior temporal gyrus. Functional connection with attachment avoidance contains critical nodes in the medial temporal lobe memory system, frontal-parietal network, social cognition, and default mode network necessary to deactivate the attachment system and inhibit attachment-related behavior.

**Conclusion and implications:**

These findings clarify the amplitude of low-frequency fluctuation and resting-state functional connectivity neural signature of attachment style, associated with attachment strategies in attachment anxiety and attachment avoidance individuals. These findings may improve our understanding of the pathophysiology of the attachment-related disorder.

## Background of the study

Social bonding conveys an intention to set up correlations with others, of that an ordinary and well-described consequence is that individual's attachment behavior. Early in life, newborns shape the first dyadic relationship experience with their caregivers, while unresponsive, neglecting, abusing, and aggressive models shape an insecure one. Specifically, there are mainly three attachment styles: secure attachment, attachment anxiety (AX), and attachment avoidance (AV). Secure attachment represents a sensitive, supportive and stable attachment pattern, and secure attachment individuals positively seek proximity and satisfaction with their relationship. However, insecure attachment generally relates to unsatisfied, distressing, and aggressive relationships; for example, attachment avoidant individuals actively avoid contact with others and distance themselves from others because they feel more comfortable being alone and suppressing their emotions. Oppositely, attachment anxiety individual exaggerates distress after separation and hyperactivates proximity-seeking strategy and high emotional expressiveness and impulsiveness (Bowlby 1969; [6, 23, 31, 41], Fraley et al. 2013).

Attachment theory posits that prior experiences of unreliable relationships can lead to deficiencies in attachment approach behavior [24]. Furthermore, numerous studies show a distinctive proximity tendency between AX and AV. Anxiously attached people worry about abandonment and seek support from others, but they perceive insufficient support [8]. Thus they name attachment figures faster in lexical decision tasks [43], remember significant proximity-related words [42], and show an attention bias towards threatening information [32]. Those avoidant attachment tendencies drive people to distance themselves from attachment figures or thoughts. Supporting this proposal is that exposed to threat avoidantly attached individuals inhibit proximity-seeking behavior, eliminate approach reaction time to secure attachment pictures in the approach-avoidance task (AAT), which spends more time to judge the direction of the picture in a picture magnifying task [66], and increase the accessibility to unfavorable attachment relevant memories [33].

Despite previous studies addressing attachment behavior pattern differences, still vital questions remain unsolved. Does the nature of the attachment behavioral distinction depend on attachment-relevant context or attachment inner working model? Is there any difference in the neural mechanism for different attachment behavior in human brain activity? How can such kinds of the method be quantified? Social bonding is not easily measurable, due to it is dynamic rather than static. Recently, several neural imaging studies on attachment seem to provide the possibility to reveal the neural mechanism underlying attachment intrinsic trait. Feldman [21] suggested that the neural system for attachment is included in three reward embodied simulation and metalizing systems. Specifically, to explore the neural mechanism for insecure attachment stimuli [59] proposed a functional neuroanatomical framework to distinguish AX from AV. He indicates that the neural basis underlying the AX is related to emotional mentalization areas such as the amygdala, insula, anterior cortex. Simultaneously, the AV is related to cognitive control areas, including the medial prefrontal cortex, precuneus, and dorsolateral prefrontal cortex.

Moreover, a multifaceted neural model of attachment proposes that there are potential neural processes underlying attachment behavior. Brain-based study findings revealed that attachment stimuli seem to activate emotional, cognitive, and motive processing brain areas [9]. As expected, both secure and insecure attachment activates an identical network that is primarily related to memory and emotional regulation, including the fusiform, middle temporal, and prefrontal areas. Attachment style has a specific behavioral pattern that affects social interaction and brain activation. The hyper-activating strategies used by anxiously attached people mainly increase stronger emotional arousal and regulation-related areas [26]. While AV reflects an ineffective social emotion regulation, close contact with the caregiver is not successful in down-regulating distress [72]. Thus, the attachment system's deactivation may activate emotional regulation areas and cognitive control-related areas [69]. There still exist specific brain areas for insecure attachment stimuli, mainly located in the insula and parietal area with distinctive functions on emotional arousal and social information [9].

However, previous brain mapping reveals the difference between the AX and AV; it is still unclear whether these specific neural patterns still exist under spontaneous brain activity? That is to say, are these specific neural mechanisms induced by attachment situation or attachment intrinsic traits as personality? Resting-state brain mapping studies indicated that spontaneous brain activity exhibited individual innate characteristics [54, 73]. It has many advantages in addressing the enduring trait's neural correlations (Yuan et al. 2014). Several studies have explored the relationship between spontaneous brain activity with AX and AV. They prove that there is a structural and functional difference between AV and AX. [49] found the AV was closely related to reduced gray matter density in the hippocampus. Correspondingly [52] explored the structural connection with attachment, and the result stated that the relation between the prefrontal and amygdala is significantly positively with attachment avoidance. According to a recent study that investigated the gray matter volume with attachment, the result showed the AV was negatively correlated with the volumes of the left middle temporal gyrus and the right Parahippocampal gyrus, while the AX correlated with that of the ventral anterior cingulate volume [71].

Since recent structural imaging studies have confirmed the difference of neural basis for AX and AV, it is necessary to explore the spontaneous neural mechanism in resting-state fMRI underlying AV and AX's trait to reveal the neural substrate of intrinsic attachment traits. The advantage for investigating resting-state brain activity are two direct indexes that are the amplitude of low-frequency fluctuation (ALFF) and functional connectivity (RSFC) [12]. One is an effective indicator to reflect the intensity of spontaneous neural activity in the brain, and the other is considered to reveal the synchrony of spontaneous neural signals in brain regions [64]. According to the secondary attachment strategy, we hypothesis that people high on attachment anxiety may amplify their activity, incredibly emotional, or cognitive monitoring-related areas. Conversely, people with a high avoidance trait may decrease the activation of emotional regulation and self-related traits but increase negative emotion and memory-related scopes.

## Materials and methods

### Participants

To achieve the study's aim, one hundred and nineteen undergraduate students at the Southwest University of China participated in the study. All participants were recruited to ensure: (1) MRI scanner compatibility; (2) right-handedness; (3) no current or past neurological, psychiatric, or cardiovascular conditions; (4) no use of medications affecting the central nervous system for six months preceding the study. Four participants were removed from the sample due to excessive head motion during data preprocessing. The remaining individuals were all young, healthy adults (64 females and 51 males; 20.81 ± 1.67 years; range: 18–24 years). After resting-state scanning, participants were required to complete the ECR-C (Experience in Close Relationship of Chinese Version) survey questionnaire, widely used to measure attachment anxiety and avoidance [35]. Before the experiment, all participants were informed of their right to privacy: they could quit the experiment at any time and be paid for their participation. All participants were told to sign an informed consent before the experiment, and the ethical committee council approved this consent of Southwest University. All the participants have the right to take part in the experiment, and they could quit the experiment at any time when they feel uncomfortable.

### Materials in attachment assessment

An instrument named the Experience in Close Relationship scale was selected to measure the level of attachment behavior. The full term of ECR is Experience in Close Relationship, which is a widely used instrument for attachment orientation [6]. This scale developed a Chinese Version of Experience in Close Relationship (Tonggui et al. 2006), performed by several large Chinese people, and proved to be a good metric indicator (Cai et al. 2014; [61]). The instrument's validity and reliability were confirmed among the Chinese population (Cai et al. 2014; [61]). This scale is a 36-item self-report questionnaire consisting of two dimensions on attachment. Eighteen items measure anxiety (e.g., "I am worried about being rejected or abandoned."), Furthermore, similar 18 items measure avoidance (e.g.," Just when my partner starts to get close to me, I find myself pulling away"). Participants were required to rate each item by indicating how they generally feel in close relationships on a Likert scale of 1(strongly disagree) to 7(strongly agree). Both subscales were reliable (Cronbach's alpha for anxiety = 0.92, and avoidance = 0.85). Anxiety (M = 4.00, SD = 1.09) and avoidance (M = 2.48, SD = 0.66) scores were not significantly correlated (r = − 0.16, p > 0.05).

### Image data acquisition

Functional imaging data obtained from a Siemens Trio 3.0 T scanner (Siemens Medical Erlangen, Germany). The echo-planar imaging (EPI) sequence with the following parameters: 32 slices; slice thickness:3 mm; TR = 2000 ms; TE = 30 ms; image matrix 64 × 64; flip angle = 90°; FOV = 220 × 220mm^2^; voxel size:3 × 3 × 3mm^3^; and 240volume.During the resting state scanning, participants were instructed to maintain an awakened state with their eyes closed, as motionless as possible, and not sleep and think about anything, in particular, resulting in a scan length of 8 min. The T1-weighed structural images were then acquired with the following scanning parameters:176 slices; slice thickness:1 mm; flip angle = 9.; TR/TE = 1900 ms/2.52 ms; FOV = 256 × 256mm^2^; voxel size:1 × 1 × 1mm^3^.

### Data preprocessing

Data were preprocessed using Dhabi and Data Processing Assistant for Resting-State fMRI [56, 57, 67]. First, we preprocessed the data using DPABI and Data Processing Assistant for Resting-State fMRI [67]. At the beginning of the resting state scanning, we excluded images from the first ten time points, while the remaining 230 volumes were left in the final data analysis. We performed slice-timing to correct slice order and realignment to adjust head motion. As a result, four participants exhibiting head motion larger than 2 mm were excluded during the formal analysis. Subsequently, we obtained the mean functional image, and the structural images co-registered with it for all participants. They were segmented as gray matter, white matter, and cerebrospinal fluid using the SPM normal tissue. All components as white matter, cerebrospinal fluid, and Friston 24 motion parameters regressed out as covariates of no interest [25]. Then each functional image was normalized to MNI space by using the normalization parameters estimated in DARTEL. Subsequently, spatial smoothing was performed with a 6-mm full-width at half maximum. Eventually, the linear trends were removed, and the images were band-pass filtered to reduce low-frequency drift and high-frequency noise [4].

### Data analysis

#### ALFF calculations

The amplitude of low-frequency fluctuations (ALFF) calculations were performed using the Resting-State fMRI Data Analysis Toolkit as previously described similarly [56, 57]. Each voxel's time series was transformed into a frequency domain with a fast Fourier transform, and the power spectrum was obtained. For the power frequency is proportional, we calculated the square root and averaged across 0.01–0.08 Hz fat each frequency of the power spectrum. This averaged square root was taken as ALFF. Subsequently, for normalization, each voxel's ALFF was subtracted from the whole brain's mean ALFF value and divided by the standard deviation to normalize the global volume effects [70]. Finally, to obtain a more accurate result, we calculated the ALFF in a gray mask, which showed higher fluctuations than that in a white mask. By using SPM12, a gray matter template for low-frequency amplitude neural signals was calculated, and the threshold was set as 0.4, and the resampling voxel size was 3*3*3 mm^3^ [14, 61, 62], all the results were performed in the gray mask.

#### Brain-behavior correlation analysis

A whole-brain analysis was performed to examine the scientific relationship between attachment and the brain's regional spontaneous functional activity. A multiple regression analysis was conducted in the SPM 12 statistics module between the mean ALFF values and AX and AV, with age and gender as a nuisance covariate. The results of multiple regression analyses were corrected using AlphaSim, which we initially implemented in AFNI software. AlphaSim correction has been used widely in rest-state fMRI studies [20, 22, 40]. The threshold was set as a corrected cluster *p* < 0.01(single voxel *p* < 0.01, cluster size ≥ 40 voxels [1080 mm^3^]). Subsequently, the mean ALFF of a cluster was extracted surviving the Alphasim correction to explore the relationship between the two attachment dimensions and these regions.

#### Functional connectivity analysis

To identify whether the regions observed in the ALFF-behavior correlation analysis work in concert with other regions as a network that correlates with attachment dimensions, the FC based on ALFF was conducted using REST 1.8 Toolkit [56, 57]. The regions significantly correlated with attachment behavior were used as seed regions of interest seeds and computed functional connectivity between the seeds and other voxels in the whole brain. After that, the functional connectivity maps have been computed in voxel-sized FC and converted to Z-maps using Fisher’s r-to z transformation. The data calculated the correlation between Z-maps and attachment dimensions through preprocessing in DPABI. Then the correlation map was conducted by AlphaSim correction (corrected cluster p < 0.01, single-voxel p < 0.01, cluster size ≥ 40 voxels [1080 mm^3^]). The significant regions defined as masks and the RSFC-behavior correlation analyses were conducted in these masks to examine whether functional connectivity strength was correlated with AX and AV.

## Results

### Behavior data

Table 1 exhibits The mean, standard deviation (SD), and range of AR-Anx and AR-Avd are listed. No significant correlation was found between the two attachment dimensions (r = − 0.16; *p* = 0.35); No significant correlations between age and AX (r = − 0.13; *p* = 0.16) or AV (r = − 0.16; *p* = 0.09); No significant gender difference in AX (t = − 0.18, *p* = 0.86) and AV (t = − 1.34, *p* = 0.18) score was observed.

**Table 1:** Male and female scores on attachment dimensions

Gender	AX			AV		
	Mean	SD	Range	Mean	SD	Range
Males (N = 51)	65.14	19.49	40–112	48.35	14.57	30–78
Females (N = 64)	65.84	22.32	24–121	53.16	22.12	25–114

*SD* Standard deviation

### Functional magnetic resonance imaging results

#### Areas of the brain in resting-state associated with attachment anxiety (AX)

**Fig. 1:**
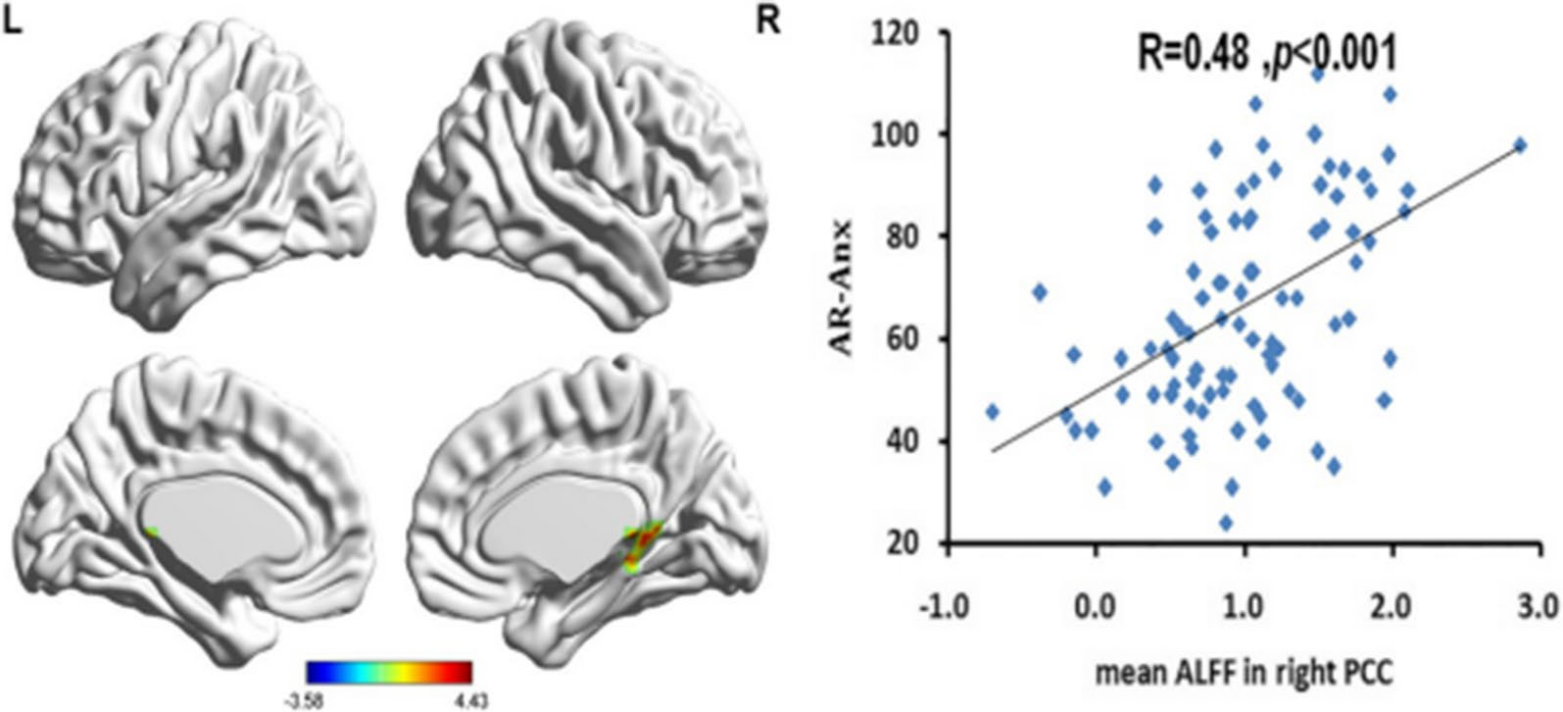
Brain regions show significant correlations between the amplitude of low-frequency fluctuations (ALFFs) and AX (the left picture). Color bars represent *t*-values. L = left, R = right. The scatter plot pictures on the right indicate significant correlations between AX and mean ALFFs in the right posterior cingulate cortex. The threshold of the corrected cluster was set at *p* < 0.01 [single voxel *p* < 0.01, cluster size ≥ 40 (1084 mm^3^)

A multiple regression analysis was performed to examine the relationship between AX and brain regions' regional spontaneous functional activists, listed in Table 2 and Fig. 1. AX was positively correlated with ALFF in the right posterior cingulate cortex (PCC) (r _cluster_ = 0.48, *p* < 0.001).

**Table 2:** Regions that showed significant correlations with AX

Region	MNI coordinates			*t*	Cluster size
	x	y	z		
Right post d cortex	3	− 42	6	4.23	40

The threshold was set at *p* < 0.01 (AlphaSim corrected: single voxel *p* < 0.01, cluster size ≥ 40 voxels [1080mm^3^]). L, left; R, right; MNI, Montreal Neurological Institute

#### Areas of the brain in resting-state associated with attachment avoidance (AV)

**Fig. 2:**
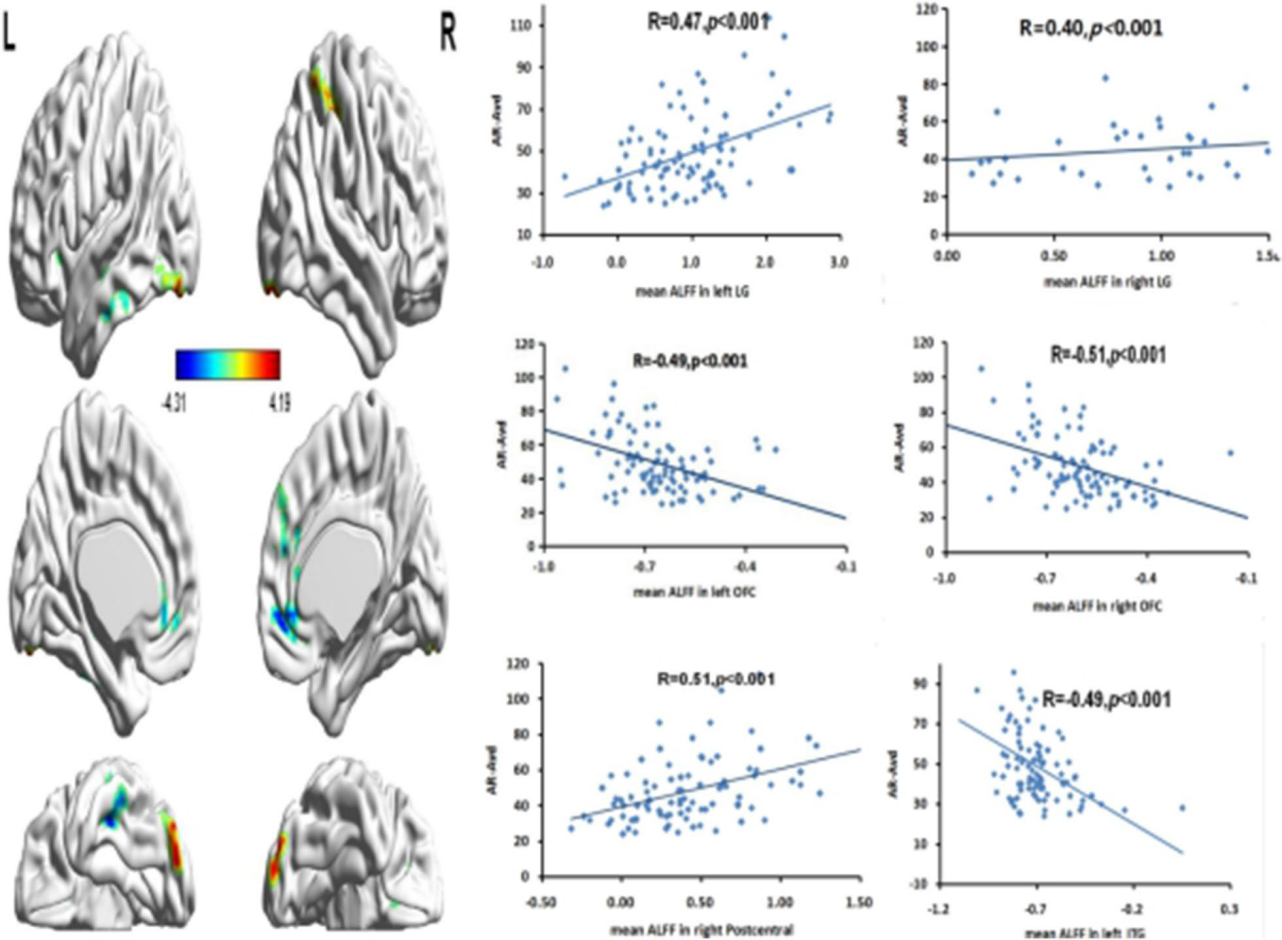
Brain regions that show significant correlations between low-frequency fluctuations (ALFFs) and AV (the left picture above). Color bars represent *t*-values. L = left, R = right. The right scatter plot pictures indicate significant positive correlations between AV and mean ALFFs in the bilateral lingual gyrus (LG) and right postcentral, and negative correlations with left inferior temporal gyrus (ITG) and bilateral orbital frontal cortex (OFC). The threshold of the corrected cluster was set at *p* < 0.01 (single voxel *p* < 0.01, cluster size ≥ 40 [1080 mm^3^])

After a multiple regression analysis, we found that AV was positively correlated with ALFFs in the bilateral lingual gyrus (LG) (r _cluster_ = 0.47, *p* < 0.001(for left LG); r _cluster_ = 0.40, *p* < 0.001(for right LG)) and the right postcentral (r _cluster_ = 0.51, *p* < 0.001), and negatively correlated with ALFFs in the left inferior temporal gyrus (r_cluster_ = − 0.49, *p* < 0.001), and bilateral orbital frontal cortex (OFC) (r _cluster_ = − 0.49, *p* < 0.001(for left OFC); r _cluster_ = − 0.51, *p* < 0.001 (for right OFC)), which are listed in Table 3 and Fig. 2.

**Table 3:** Regions that showed significant correlations with AV

Region	MNI coordinates			*t*	Cluster size
	x	y	z		
Positive correlation					
Left lingual gyrus (LG)	− 30	− 87	− 18	4.19	84
Right lingual gyrus (LG)	24	− 92	− 16	3.83	42
Right postcentral	42	− 39	57	4.03	51
Negative correlation					
Left Orbital Frontal cortex (OFC)	− 18	24	− 12	− 3.63	79
Right Orbital Frontal cortex (OFC)	21	36	− 9	− 4.31	126
Left Inferior Temporal gyrus (ITG)	− 42	− 15	− 21	− 4.23	134

The threshold was set at *p* < 0.01 (AlphaSim corrected: single voxel *p* < 0.01, cluster size ≥ 40 voxels[1080mm^3^]). L, left; R, right; MNI, Montreal Neurological Institute

#### Functional network associated with AX and AV

Functional connectivity analysis was performed to investigate whether the brain regions observed in the previous analysis function synergistically with other regions. The connectivity between the right posterior cingulate cortex and right fusiform gyrus was positively correlated with AX; meanwhile, the connection between the bilateral inferior temporal gyrus (ITG), the bilateral OFC, the right postcentral gyrus, and other regions distributed in the critical nodes of the frontotemporal network, all these connections were positively correlated with AV. These results are shown in detail in Tables 4 and 5.

**Table 4:** Functional network with AX

Brain Region	MNI coordinates			*cluster*	_*speak*_	_*cluster*_
	x	y	z			
R post d cortex as the seed						
Fusiform-R	38	− 75	− 15	69	.37	.40^***^

The threshold was set at *p* < 0.01(AlphaSim corrected: single voxel *p* < 0.01,

cluster size ≥ 40 voxels[1080mm^3^]). L, left; R, right; MNI, Montreal Neurological Institute

**Table 5:** Functional network with AV

Brain region	MNI coordinates			*cluster*	_*speak*_	_*cluster*_
	x	y	z			
L Lingual as the seed						
None significant						
R Lingual as the seed						
None significant						
L Inferior Temporal Gyrus as the seed						
Inferior Temporal Gyrus-R	39	0	− 33	306	.43	.45^***^
L Frontal-Med-Orb as the seed						
Frontal-Sup-L	− 18	24	60	695	.41	.50^***^
Frontal-Inf-L	− 42	33	3	127	.38	.40^***^
Frontal-Inf-R	54	33	3	165	.39	.40^***^
Temporal-Mid-R	51	− 51	9	43	.35	.35^***^
Temporal-Inf-R	45	3	− 36	234	.42	.45^***^
Lingual-L	− 21	− 63	3	43	.37	.35^***^
Insula-R	45	− 6	− 3	48	.36	.40^***^
Cuneus-R	15	− 72	24	190	.41	.38^***^
R Frontal-Med-Orb as the seed						
Frontal-Sup-R	12	57	33	85	.38	.43^***^
Frontal-Sup-L	− 15	27	60	816	.45	.47^***^
Frontal-Mid-L	− 42	12	48	121	.40	.44^***^
Temporal-Mid-L	− 36	18	− 33	571	.43	.49^***^
Parahippocampal-R	33	− 9	− 27	125	.40	.43^***^
R Postcentral as the seed						
Temporal-Inf-L	− 39	0	− 33	195	.42	.43^***^
Parietal-Inf-R	33	− 51	42	2328	.51	.47^***^
Frontal-Inf-R	48	18	21	647	.46	.49^***^
Frontal-Inf-L	− 42	33	9	415	.49	.51^***^
Cuneus-L	− 12	− 87	15	912	.41	.40^***^

The threshold was set at *p* < 0.01(AlphaSim corrected: single voxel *p* < 0.01, cluster size ≥ 40 voxels [1080mm^3^]). L left; R, right; MNI, Montreal Neurological Institute.; *r*_*peak*_ stands for the correlation coefficient between the connective strength of the peak point and the seed with the behavior; _cluster_ stands for the correlation coefficient between the mean value of the peak point and the seed with the behavior

## Discussion

Our study intended to investigate the neural bases of attachment anxiety and avoidance in a resting-state fMRI environment. The results showed that people high on attachment anxiety or attachment avoidance exhibited different neural activity patterns in emotion-, cognition- and memory areas. Specifically, the AX was statistically significantly positively correlated with the ALFF in the right posterior cortex (PCC). AV positively correlated with the ALFF in the bilateral lingual gyrus (LG) and right postcentral gyrus but negatively correlated with ALFF in the bilateral orbital frontal cortex (OFC) and left inferior temporal gyrus (ITG).

### Discovering the relation between ALFF and AX

The posterior cingulate cortex is the central area for emotional processing, where the anterior part is specialized for affective perception and intensity. In contrast, the mid-and post- part is responsible for affective evaluation [11]. This study found the ALFF in PCC was significantly correlated with the level of AX. As a precede hypothesis, people high on attachment tend to exaggerate threatening stimuli and maintain a clingy intimacy with others. Thus, they may over-evaluate emotional intensity and unintended outcomes, which may increase the value of PCC, implement emotional appraisal, and search for possible threatening stimuli [16]. The rating scores on self-trait were negatively related to attachment anxiety and increased PCC activation during the self-appraisal.

### Discovering the relation between ALFF and AV

The result demonstrates that AV is positively correlated with ALFF's value in bilateral LG and right postcentral gyrus but negatively correlated with that of the bilateral OFC and left ITG. Ortigue [47] found that bilateral LG was associated with memory retrieval of attachment figures and abstract representations of others. Highly attached avoidant people often keep themselves away from close ones, as they retrieval more misery memory about interaction with caregivers, which supports their negative-other models. To avoid suffering from close people, they downplay the need for proximity (Mikunlincer 2007). Thus it can be concluded that the value of ALFF in bilateral LG will increase with the scores of AV. Another ALFF region linked to the AV is the postcentral gyrus, which plays a vital role in motor control and generating somatic sensation [36]—as compared with a loved-one, imaging a stranger's hands or feet in painful increased activity in the temporal-parietal junction (TPJ) [13]. Likewise, when subjects were processing social emotions from a third-person perspective, a cluster in the postcentral gyrus was detected. That implies that the postcentral gyrus played a significant role in self-other distinction, viewing close others as much as a stranger [53]. In this study, ALFF's value in the postcentral gyrus is related to the avoidant tendency from interaction with others.

On the contrary, AV was negatively related to bilateral OFC and left ITG. OFC is a vital part of the neural network involved in emotional regulation [28]. AV is insufficient in processing affection and requires more cognitive control on attachment-related thoughts [63]. OFC's activation was moderated by the level of attachment avoidance, which decreases the degree of activation for people high on attachment avoidance during emotional regulation unsuccessfully [50]. Besides, the OFC is associated with top-down cognitive processes, which affect the interpretation of stimuli and expectations about outcomes [46]. Highly avoidant people have an adverse explanation of others' attachment behavior and show pessimistic expectations for a close relationship. In this way, they are more likely to fail to suppress emotion and implement the reappraisal process [17, 60].

Apart from OFC, the finding noted that ALFF's value in ITG has a significant negative influence on AV. The ALFF of ITG was positively related to self-confidence because it stores self-related knowledge. The higher the value of ITG is, the more self-knowledge is. Security people provide people with an increased ability to regulate their emotions and behavior, this experience-rich Oneself mental resources, and their high self-confidence [27]. The value of ALFF in ITG increases with self-esteem [14].

Conversely, people high on avoidant seem to have high self-confidence, the same as securely attached people. Still, they often overestimate their ability to be independent and minimize their vulnerability of being close to others. Therefore, it implies that they lack self-knowledge, so the value of ALFF in ITG decreases with AV scores.

### The FC between regions activated in ALFF and AX

Our study's fundamental finding stated that the functional connectivity (FC) between right PCC and fusiform (FFA) was enhanced with the AR-Anx scores. As FFA was sensitive to facial expression, facilitating emotional detection switches to external attachment behavior such as searching attachment figures ([10]; Valentinos et al. 2018). [15] adopted a morph movie paradigm on facial expression to explore emotion sensitivity for anxiously attached people. The result stressed that facial processing was elaborated, and earlier for anxious attachment, the individual was more likely to perceive the change of facial expression earlier than other people. Emotional detection and exaggerate emotional outcomes are typical characteristics, the individual fear being abandoned by the partner continually, especially alert to the threat from visual perception surrounding (McWilliams et al. 2007). In line with our study, a resting fMRI study revealed the FC between bilateral occipital lobes increased with attachment anxiety scores, which strengthened visual searching and attention alertness on attachment-related information [51]. The FC in our study observed individuals with insomnia symptoms or borderline personality, which implied that emotional dysfunction is linked to excessive emotional disaster [18, 37].

### The FC between regions activated in ALFF and AV

Functional connectivity makes the relationship between several brain areas' time-series as an indicator, demonstrating the degree of brain function integration. In this study, we have explored the FC related to the AV scores. As we hypothesis previously, high on avoidant people are associated with the regulatory, top-down process. Because of their negative models of others, highly avoidant people recruited more self-knowledge areas [27], speculating others' thoughts, and retrieving memory [60].

There is no connection related to the bilateral lingual gyrus and the AV. Nevertheless, the FC of the bilateral inferior temporal gyrus increases with AV scores. This connection is a vital component of the medial temporal lobe (MTL) system [19]. Being activate in episodic memory may retrieve more negative knowledge about attachment experience. Likewise, the higher the AV score, the more functional connectivity between bilateral ITG [5]. Front polar areas and parietal lobe were associated with avoidant attachment since they were related to a self-other distinction, not overlap [44, 45, 53]. We have found that the FC between bilateral OFC and frontal–temporal areas were positively correlated with the AR-Avd. Specifically, bilateral OFC is coupling with the bilateral superior frontal gyrus (SFG), inferior frontal gyrus (IFG), middle temporal gyrus (MTG), left middle frontal gyrus (MFG), and right inferior temporal gyrus (ITG). These regions are constituted of the mentalizing network, supporting the conceptualization of attachment mentalization. The avoidant individual speculates others' thoughts and subsequent behavior, activating more areas in the frontal and temporal lobe. A study indicated that the FC between the caudate nucleus (CN) and temporal regions decreased after presenting the insecure-dismissing narrative [34]. Mentalizing other's thoughts in negative other model provide avoidant people reasonable explanation to avoid intimacy and closeness.

Besides, research on executive control shows that the IFG is part of a front parietal attention network and plays an essential role in cognitive inhibition ([3], Aron et al. 2014, [29]), so IFG plays a controlling role on attachment-related stimuli (Harri et al. 2000). A meta-analysis recently discussed multiple neural correlations of attachment style from the structural and functional differences in brain areas. The result showed significant differences in the IFG, which deactivated attachment-related stimuli processing and memory from one’s early attachment experience [48, 50]. Besides, the FC between parahippocampal and OFC was increased with the AV scores, which was related to episodic memory; this connection strengthened the relationship between affection processing and memory storage [2, 58]. The FC between OFC and insula, cuneus, was related to dysfunction in emotional regulation among BPD or PTSD disorders. As a result, it may represent an inefficient emotional regulation (Yan et al. 2011; [65]).

Based on the postcentral gyrus as the seed of FC, bilateral IFG, ITG, IPL, and cuneus were coupling with the postcentral gyrus; all these areas were critical nodes of the default mode network (DMN). Neural imaging studies suppose that DMN functions as an internal psychological mediator, involving in processing theory of mind, mental time travel, autobiographical memory, mind wandering, and so on [38]. Externalizing social withdrawal or inhibiting proximity-seeking behavior may elicit more mental representation of unreliable relationships, supporting avoidantly attached people to keep themselves away from potential intimacy and resulting in excessive spontaneous activity of DMN. The default mode network is supposed to play a crucial role in brain functional integration; participants with perceived dysfunctional parenting (PDP) exhibited increased DMN connectivity after the activation of attachment memories. Exposed to misery, parenting memory leads to a transitory failure of functional integration and consequent mentalization disturbance [1].

This study explores the spontaneous brain activity underlying attachment behavior; the ALFF and RSFC are useful indicators to reveal attachment traits' neural basis. Specifically, the AX is closely associated with ALFF in the right PCC, over-estimating emotional intensity and exaggerating outcomes. Furthermore, the FC between PCC and FFA increases detection ability for potential threat or separation information, facilitating behavior motivation. Comparatively, the brain areas correlated with attachment avoidance are complicated. The AV is positively correlated with the ALFF in bilateral LG and right postcentral but negatively with bilateral OFC and ITG. As discussed in the previous part, the activation areas positively related to attachment avoidance can lead to more negative memory retrieval and unreliable others' representation, which holds them back into the attachment system and shows more deficiency in the capacity to benefit from attachment. The value of ALFF in OFC and ITG decreases with attachment avoidance scores because they are deficient in emotional regulation and lack sufficient self-knowledge. Functional connection with AV contains key nodes in the MTL memory system, frontal-parietal network, social cognition, and default mode network. These connections are necessary for deactivating the attachment system and inhibiting attachment behaviors. Autobiographic memory on attachment strengthens a sense of distrust in attachment figures, negatively mentalizing others and suppressing negative thoughts about themselves. Thus they tend to downplay the need for intimacy and distance themselves during stress or threat so that they can benefit less from the attachment system.

## Conclusion

Resting-state fMRI was exploited as a predictive indicator for individual differences in personality, attachment dimensions, and personality differences in social bonding [41]. We explored the ALFF and RSFC underlying attachment dimensions to explain attachment trait differences presenting in attachment behavior. The result shows that the value of ALFF in PCC increases with AX's level, reflecting the excessive evaluation of attachment stimuli and supports the need for proximity. FC between PCC and FFA facilitates searching for threat or stress information to maintain a hyper-activation attachment system. On the other side, ALFF's value in bilateral LG and right postcentral gyrus increases with higher AV, while bilateral OFC and ITG decrease. These regions are related to mentalize others, retrieve attachment experience, store self-knowledge, and regulate negative emotions. FC synchronizing with these regions constitutes the MTL memory system, social cognition network, frontal–temporal network, and default mode network. These connections are involved in excessive mentalizing others, self-other representation distinction, and negative emotion inhibition; this may lead to insufficiency for brain functional integration. The clinical study pointed out that lagged DMN functional communication was associated with symptoms of schizophrenia in a resting state [55]. Thus it is necessary to clarify the relationship between these brain networks and attachment avoidance in the future; high on attachment avoidance may be associated with brain integration dysfunction.

To compare the result of our study and other similar neural researchers, we collected some neural mechanisms under attachment, and the result was concentrated on two central brain regions. The one is the amygdala (Rigion et al. 2016), of which function is processing emotional stimuli and information. The other region is the inferior frontal gyrus (IFG), whose function controls one’s cognition or inhibits attachment-related thoughts or memories [48]. These results were not the same as our study, and one reasonable explanation is that the amygdala is more likely to be activated in task-induced fMRI, while the IFG was synchronized with attachment avoidance. Therefore, these results indicated that spontaneous brain activity underlying attachment trait has a unique neurobiological signature. The future study could be concerned with RSFC between attachment and these two regions.

Similarly, the social aversion network was supposed to be linked to dismissing attachment narratives; the FC between the dorsal anterior d cortex and middle temporal gyrus increased after listening to it (Linda et al. 2016). On the contrary, the social approach network includes the bilateral caudate nucleus (CN), increased functional connectivity with temporal-parietal junction (TPJ), posterior cortex (PCC) after the presentation of attachment anxious narrative [34]. Overall, Vrticka et al. [59] explained the neural circuit as an emotional and cognitive mentalizing neural basis. This study supported the hypothesis that the AV and AX recruits "top-down" and "down-top" neural circuits for different attachment strategies. Potential neural mechanisms helped us understand attachment behavior differences in human bonding.

## Data Availability

All datasets collected from our participants can be provided based on the request.
